# Endoclip and snare combined traction and resection for treating gastric submucosal tumor with extraluminal growth

**DOI:** 10.1055/a-2765-5886

**Published:** 2026-01-22

**Authors:** Jia Xu, Zhouyue Zhang, Muhan Lü, Xiaowei Tang

**Affiliations:** 1 556508 Department of Gastroenterology, The Affiliated Hospital of Southwest Medical University, Luzhou, P. R. China

Endoscopic resection of extraluminal gastric submucosal tumors is technically demanding due to their location outside the gastric lumen, which often limits visualization and access. Conventional endoscopic techniques often fall short in addressing these challenges, frequently requiring traction-assisted innovative methods to ensure safe and complete resection 1 2 3 4 . We describe a novel method using a combined endoclip and snare to perform endoscopic full-thickness resection (EFTR), successfully completed in approximately 60 minutes.

**Fig. 1:**
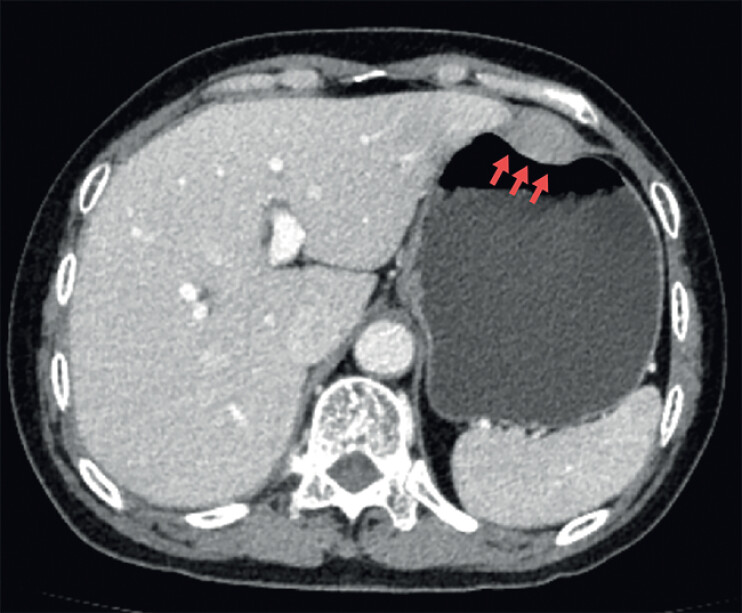
Preoperative computed tomography image showing a 3.5 × 2.5 cm extraluminal mass (arrow) along the greater curvature of the stomach.

**Fig. 2:**
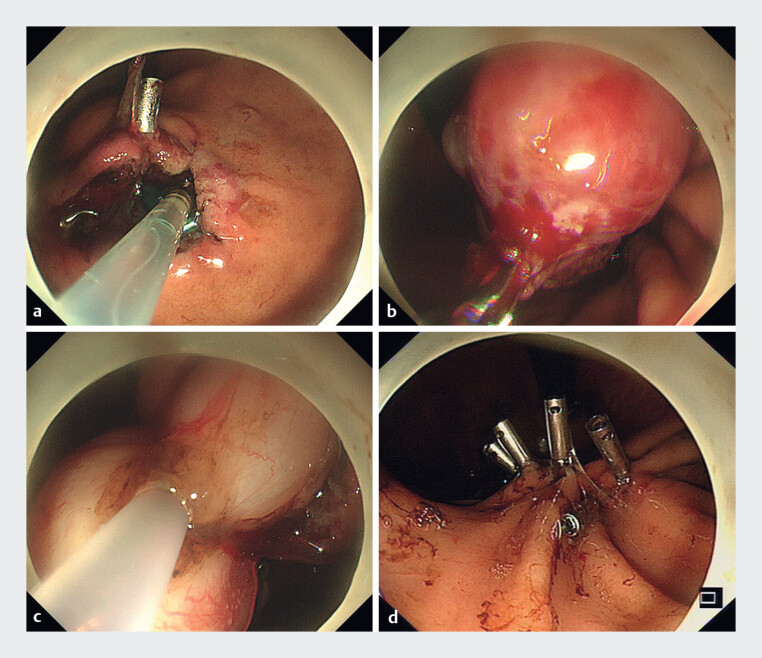
Endoscopic procedure steps for full-thickness resection of the extraluminal gastric submucosal tumor. **a** Full-thickness incision made adjacent to the tumor. **b** An endoclip was used to grasp and retract the tumor into the gastric lumen, forming an artificial pseudopedicle. **c** A snare was positioned around the base of the pseudopedicle. **d** The gastric wall defect was closed with multiple endoclips after resection.

**Fig. 3:**
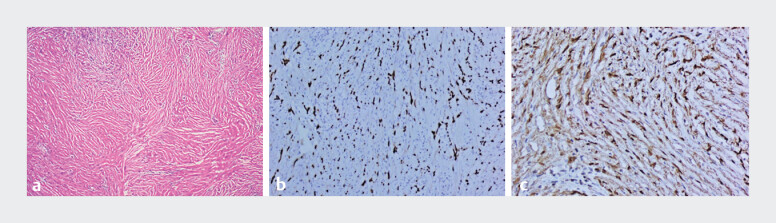
Histopathological results of the resected gastric lesion. **a** Hematoxylin and eosin (H&E) staining reveals a typical proliferation of spindle-shaped tumor cells. **b** Immunohistochemical stain for SOX10. **c** Immunohistochemical stain for S-100.

A 60-year-old female patient presented with epigastric discomfort. Computed tomography scanning revealed a 3.5 × 2.5 cm extraluminal mass located along the greater curvature of the stomach ( Fig. 1 ). Endoscopic examination identified a hemispherical submucosal bulge in the upper gastric body. Given the lesion's substantial size, symptomatic presentation, and potential malignant risk, EFTR was indicated. The procedure was conducted under general anesthesia with endotracheal intubation in a standard endoscopy suite, which involved four steps ( Video 1 ). A full-thickness incision was made adjacent to the tumor using a DualKnife ( Fig. 2 **a** ), creating an opening adequate for tumor passage. An endoclip was deployed to grasp and pull the tumor into the gastric lumen, forming an artificial pseudopedicle ( Fig. 2 **b** ). A snare was placed securely around the base of this pseudopedicle ( Fig. 2 **c** ). The lesion was resected by cutting along the snare, and the resultant gastric wall defect was carefully closed using multiple endoclips ( Fig. 2 **d** ). Histopathology confirmed schwannoma, with spindle cells on H&E ( Fig. 3 **a** ) and positive SOX10/S-100 immunostaining ( Fig. 3 **b, Fig. 3 c** ).

Demonstration of endoscopic procedure.Video 1

This endoclip-snare traction technique advances management of extraluminal gastric submucosal tumors. By enabling effective traction and stabilization of the tumor, the method facilitates safe resection and simplifies closure, thereby overcoming a major limitation of conventional EFTR. It shows promise for broader application in resection of similarly challenging lesions and underscores the value of instrumental innovation in advancing endoscopic surgery.
